# What Is Redundancy?

**DOI:** 10.3390/e28020167

**Published:** 2026-02-01

**Authors:** Clifford Bohm, Christoph Adami, Arend Hintze

**Affiliations:** 1 Department of Computer Science and Engineering, Michigan State University, East Lansing, MI 48824, USA; 2 Department of Microbiology, Genetics, and Immunology, Michigan State University, East Lansing, MI 48824, USA; adami@msu.edu; 3 Program in Evolution, Ecology, and Behavior, Michigan State University, East Lansing, MI 48824, USA; 4 Department for MicroData Analytics, Dalarna University, 791 88 Falun, Sweden; ahz@du.se

**Keywords:** information decomposition, redundancy, synergy

## Abstract

Redundancy is a central yet persistently ambiguous concept in multivariate information theory. Across the literature, the same term is used to describe fundamentally distinct phenomena. Operational redundancy concerns how different inputs relate to the prediction of output states, while informational redundancy concerns content overlap among inputs relevant to an output. These notions are routinely conflated in decompositions of mutual information, leading to incompatible definitions, contradictory interpretations, and apparent paradoxes—particularly when inputs are statistically independent. We argue that the difficulty in defining redundancy is not primarily technical, but conceptual: the field has not converged on what redundancy is meant to signify. We formalize this distinction by identifying two classes of redundancy. Operational redundancy encompasses task-relative properties and covers conditions when inputs are sufficient or substitutable for prediction. Informational redundancy concerns shared content among inputs, grounded in mutual information between them. Using functional examples and biased input ensembles, we demonstrate the practical distinction between these classes: inputs with no informational overlap can exhibit operational redundancy, while partial observation can induce statistical correlations that create content overlap without reflecting the underlying functional structure. We conclude by proposing a clear separation of these concepts and outlining minimal commitments for each. This separation clarifies why redundancy remains elusive, why no single measure can satisfy all intuitions, and how future work can proceed without redefining information itself.

## 1. Introduction

The decomposition of mutual information into non-overlapping, positive, and human-interpretable components has become a central objective in contemporary information theory, with applications ranging from neuroscience and complex systems to machine learning and fairness analysis [1,2,3]. The most popular proposal for information decomposition is Partial Information Decomposition (PID) [4], which suggests that information can be decomposed into “unique”, “synergistic”, and “redundant” components. However, despite substantial technical progress, the concept of redundancy remains unsettled [5,6,7]. Multiple formal definitions coexist, often yielding incompatible results even for simple systems, indicating that disagreement persists regarding what redundancy is meant to represent and how it should relate to Shannon information measures [8,9,10].

The difficulty in defining redundancy is not primarily technical but conceptual: the field has not converged on which phenomena the term is meant to capture. In particular, notions relating to predictive capacity and informational overlap have been repeatedly conflated under the single label of redundancy. To clarify, we introduce operational redundancy and informational redundancy to label these two classes.

Operational redundancy, in particular, admits many formalizations, depending on how one chooses to characterize relevance, sufficiency, or substitutability. Rather than attempting to exhaust this space, we focus on two illustrative examples—conditional irrelevance (CIR) and predictive redundancy (PR)—which demonstrate how operational properties manifest in different contexts. In contrast, informational redundancy (IR) corresponds to overlap in information content among inputs. Together, these definitions serve to delineate the conceptual landscape rather than to fix its boundaries.

A key source of confusion is the implicit assumption that any term identified as redundancy is a valid element of a decomposition of mutual information; that is, that it should correspond to a well-defined component of such a decomposition. However, many uses of the word redundancy in practice are operational and do not correspond to elements of an information decomposition. For example, redundancy may be invoked to describe sufficiency relative to a task (any one of several variables is enough to predict an outcome), or irrelevance under conditioning (once one variable is known, another can vary freely without affecting the outcome) [7,8]. These notions are operationally meaningful, but they are not reducible to mutual information terms.

Recent work on information fragmentation [11] illustrates this distinction in applied settings, where multiple variables may independently predict the same feature without sharing mutual information among themselves, and where conditional irrelevance emerges as a property of observed data rather than of an underlying functional specification. Such cases demonstrate that predictive overlap, conditional necessity, and shared informational content correspond to different structural relationships, even though all are colloquially labeled as “redundancy.”

Our goal is not to propose a new partial information decomposition, nor to modify existing frameworks, but to clarify the conceptual landscape in which such decompositions are interpreted. By disentangling operational from informational redundancy, we aim to explain why redundancy has proven difficult to formalize, why disagreements between measures are often unavoidable, and why certain intuitively “redundant” systems necessarily yield zero redundancy in Shannon-based decompositions. This reframing suggests that the primary challenge is not selecting the correct redundancy measure, but first identifying which notion of redundancy—operational or informational—is appropriate for the question being asked.

## 2. Formalizing Operational vs. Informational Redundancy

In this section, we formalize two fundamental classes of redundancy: operational and informational. Operational redundancy encompasses operational properties that describe predictive relationships between inputs and outputs—whether inputs are necessary, sufficient, or interchangeable for prediction. We illustrate this class through two representative examples: Conditional Irrelevance (CIR) and Predictive Redundancy (PR). These are not independent theoretical constructs, but rather complementary manifestations of operational properties within the same conceptual class. In contrast, Informational Redundancy (IR) is a structural property that describes shared content among inputs themselves, grounded in mutual information between them.

The definitions that follow reflect this fundamental divide. CIR and PR can be stated concisely because they describe straightforward relationships between variables and outcomes in observed data. IR, however, requires substantial development because it confronts a core limitation of classical Shannon information theory: mutual information quantifies statistical dependence but does not directly isolate shared content.

### 2.1. Formalizing Operational Redundancy via CIR and PR

#### 2.1.1. Conditional Irrelevance (CIR)

Let $X=\{X_{1},X_{2},\ldots ,X_{n}\}$ be inputs, *Y* be an output, and let *D* be a dataset of observed (X,Y) pairs.

$X_{i}$ is conditionally irrelevant to *Y* with respect to *D* if the following applies:

(1) $$I(Y;X_{i}|X_{-i})=0$$

where $X_{-i}=X\backslash \left\{X_{i}\right\}$ and the mutual information is computed using the empirical distribution from *D*.

When this equality holds, the input $X_{i}$ provides no additional information for predicting *Y* beyond what is contained in $X_{-i}$, given the support of the observed data. CIR describes whether an input matters for predicting the output within the context of the observed data. It is a statement about predictive necessity under conditioning, not about informational content.

We use “CIR” rather than “CI” to avoid confusion with Conditional Independence. Conditional independence is a property of a probability distribution. CIR is an empirical property of a specific dataset—a variable may be CIR in *D* even if it is not conditionally independent in the underlying population distribution.

In plain English, $X_{i}$ is conditionally irrelevant if varying it doesn’t affect the output, given the states of other inputs.

#### 2.1.2. Predictive Redundancy (PR)

Let $X=\{X_{1},X_{2},\ldots ,X_{n}\}$ be inputs, *Y* be output, and let *D* be a dataset of observed (X,Y) pairs.

A set R⊆X with |R|≥2 is predictively redundant for *Y* if the following applies:

(2) $$\forall R_{i}\in R,\;I\left(Y;R_{i},X_{-R}\right)=I\left(Y;X\right)$$

where $X_{-R}=X\backslash R$.

PR describes whether inputs are interchangeable for prediction. If a set of variables are PR, then each member is CIR on its own. Like CIR, PR is a statement about predictive sufficiency, not about shared informational content.

In plain English, all members of *R* are interchangeable—any one will suffice for predicting *Y* when combined with $X_{-R}$.

### 2.2. Formalizing Informational Redundancy (IR)

The preceding definitions formalize operational redundancy—properties of predictive relationships between inputs and outputs. CIR evaluates whether an input is necessary under conditioning; PR evaluates whether inputs are interchangeable for prediction.

Informational redundancy is fundamentally different. IR quantifies shared content among inputs that contribute to an output. It measures, in bits, the portion of information about Y that arises from informational content that two or more inputs hold in common. This is not a statement about predictive relationships—it is a statement about the structure of information itself.

Constructing such a measure requires confronting a limitation of classical information theory: while mutual information quantifies dependence between variables, it does not isolate shared content. The remainder of this section develops the formal apparatus needed to do so.

#### 2.2.1. Common Information and Its Classic Limitations

For two random variables $X_{1}$ and $X_{2}$, mutual information $I(X_{1};X_{2})$ quantifies the degree of statistical dependence between them. However, mutual information does not identify the structure or location of the shared information, nor does it isolate a distinct object corresponding to that shared content. Shannon deliberately excluded semantic or content-related considerations from the scope of information theory, leaving the isolation of shared content as an open problem.

Several notions of common information have been proposed, most notably the Gács–Körner common information [12], Wyner common information [13], and information bottleneck methods [14]. These approaches attempt to extract a shared component between random variables, typically by identifying deterministic or nearly deterministic common structure. It is well understood [12], however, that such measures are necessarily approximations and for general joint distributions, no classical random variable can fully represent all of the content quantified by $I(X_{1};X_{2})$. This reflects a fundamental limitation of classical probability theory rather than a deficiency of these measures.

We explicitly accept this limitation. The framework developed here addresses it by working with idealized constructs that capture shared content exactly by definition, without requiring classical representation.

#### 2.2.2. An Idealized Common-Information Object

To reason precisely about shared content, we introduce an idealized notion of common information. For two random variables $X_{1}$ and $X_{2}$, we define an object *C* by the information-theoretic constraints:

(3) $$H\left(C\right)=I\left(X_{1};X_{2}\right)=I\left(C;X_{1}\right)=I\left(C;X_{2}\right).$$

These equations define *C* as an object that contains exactly the information shared between $X_{1}$ and $X_{2}$, no more and no less. While these constraints uniquely specify the informational properties of *C*, for general joint distributions, no classical random variable can satisfy them simultaneously [12]. The idealized framework introduced here is therefore not a choice of convenience but a logical necessity: if shared content is to be formally defined, it must be defined through information-theoretic constraints rather than classical representation. Whether such objects admit non-classical realization is an open question, but the framework itself is required to reason precisely about informational redundancy.

We interpret *C* as existing in what might be called a super-informational state—defined entirely by the constraints it must satisfy, independent of any particular realization. For this framework to operate, *C* must possess certain minimal properties: it must be capable of storing state constraints, it must have a well-defined entropy, and it must be able to enter into mutual information relationships with random variables. These are the requirements for *C* to participate in information-theoretic analysis. We are not proposing a specific form for *C*, nor claiming to know how such an object might be realized, if at all. The framework depends only on *C* having these information-theoretic properties. That the common-information object is not currently operational does not diminish its status within a correct information-theoretic model: its explanatory role is fixed by the constraints it satisfies, not by the availability of a representation.

#### 2.2.3. The Common-Information Operator

We generalize this construction by introducing a common-information operator C(·), which maps a collection of variables to their shared content. The operator is assumed to satisfy the following properties:

- Order invariance (Symmetry): The order of inputs does not affect the resulting common information. All inputs are treated equivalently.
- Monotonicity: Adding additional variables can only reduce (never increase) the shared content.

Intuitively, each additional variable removes any portion of the current common information that that variable does not itself possess.

For two variables, we write the following:

(4) $$C=C(X_{1},X_{2}).$$

For more than two variables, common information is constructed iteratively. Let

(5) $$C_{1}=C\left(X_{1},X_{2}\right),$$

and for each additional variable $X_{i+1}$, define the following:

(6) $$C_{i+1}=C\left(C_{i},X_{i+1}\right).$$

Because the operator is order-invariant, this construction defines a common-information object relative to any subset of inputs, independent of the order in which they are combined. Thus, $C(X_{1},X_{2},X_{3})$ is well-defined whether computed as $C(C\left(X_{1},X_{2}\right),X_{3})$, $C(X_{1},C\left(X_{2},X_{3}\right))$, or $C(C\left(X_{1},X_{3}\right),X_{2})$.

#### 2.2.4. Identifying Content Overlap

Informational Redundancy concerns shared content with respect to an output *Y*. To construct IR, we must first identify its atomic components: for each subset of inputs (of size two or more), we isolate what content those inputs share with each other and with *Y*.

We introduce a general notation for these overlap objects. For any subset *S* of input indices, $O_{S}$ denotes the common information among those inputs and the output:

(7) $$O_{S}=C\left(X_{S},Y\right),$$

where $X_{S}=\left\{X_{i}:i\in S\right\}$. For instance, the following:

(8) $$\begin{array}{cc} O_{1} & =C(X_{1},Y), \end{array}$$

(9) $$\begin{array}{cc} O_{13} & =C(X_{1},X_{3},Y), \end{array}$$

(10) $$\begin{array}{cc} O_{1234} & =C(X_{1},X_{2},X_{3},X_{4},Y). \end{array}$$

Each $O_{S}$ represents an atom of relevance—the content shared by all inputs in *S* that is also relevant to *Y*. These atoms are the building blocks from which IR is constructed.

#### 2.2.5. Informational Redundancy via Inclusion–Exclusion

Each overlap object captures common information present in different subsets of the system. Since IR measures shared content among multiple inputs, we might begin by examining pairwise overlaps such as $O_{12}$ and $O_{13}$. However, these pairwise objects may themselves share content with each other—content that would be counted multiple times if we simply summed their entropies. Conversely, we cannot rely solely on higher-order objects, like $O_{123}$, as these capture only what all inputs in the subset share, potentially excluding content that is shared between specific pairs but not the full set.

Thus, neither examining pairwise overlaps alone nor focusing exclusively on higher-order overlaps is sufficient. To isolate irreducible components of shared, output-relevant content, we must systematically account for all levels of the subset structure while avoiding overcounting. This is accomplished through inclusion–exclusion principles, formally realized as Möbius inversion on the subset lattice of inputs [15] (Readers may recognize this inclusion–exclusion structure from other information-theoretic measures such as interaction information and O-information. However, the similarity is purely algebraic: while those measures apply inclusion–exclusion to entropies of the original variables and their combinations, here it is applied to entropies of common-information objects).

IR is not defined by preference or constructed to satisfy axioms. It answers a specific question: how much of I(X;Y) arises from content shared among multiple inputs? Within the common-information framework, this question has a determinate answer: IR measures the overspecified portion—information present in more than one input that contributes to Y.

For two inputs, the IR content is simply as follows:

(11) $${\mathrm{IR}}_{12}=O_{12}.$$

For three inputs, the inclusion–exclusion correction becomes as follows:

(12) $${\mathrm{IR}}_{123}=O_{12}+O_{13}+O_{23}-2O_{123}.$$

For four or more inputs, the expressions grow correspondingly more complex.

In plain English, information redundancy (IR) measures overspecification—information about Y that is present in multiple inputs (at least two) simultaneously.

## 3. Examples

### 3.1. Understanding Conditional Irrelevance

*Bob was let go after the merger because his position was redundant. Later, Bob heard that the company was scrambling because Alice quit. “I guess I wasn’t that redundant after all.”*

We can assume that both Bob and Alice can be in the states employed and unemployed, and the company can be in the states operational and failing. In this context, Bob’s CIR is formally evaluated as follows:

(13) I(Company;Bob∣Alice=employed)=0.

That is, given that Alice is employed, Bob’s state has no effect on the operational status. Under this condition, Bob is deemed unnecessary, and his position is eliminated. This is exactly a statement of conditional irrelevance: Bob’s state can vary freely without affecting the company’s operations, provided Alice is employed.

Importantly, this judgment is explicitly conditional. It does not assert anything about Bob’s general relationship to the operational status, nor does it imply that Bob and Alice play interchangeable roles. It merely reflects that, under a specific condition that holds at the time of evaluation, Bob carries no additional informational value about operational status.

When Alice later quits, the conditioning context changes. The previous conclusion about Bob no longer applies, not because the original assessment was incorrect, but because his irrelevance was conditional on circumstances that no longer obtain.

CIR need not be symmetric. Let us assume that Bob is an accountant, and Alice is an accountant and a notary, and that the company requires both to operate. In this case, Bob is CIR on the assumption that Alice works for the company, whereas Alice is not CIR under the same assumption about Bob. If there were another notary on staff, perhaps Tom, then, on the condition that Bob and Tom are both employed, Alice would be CIR.

Formally, Alice’s CIR can be expressed as follows:

(14) I(Company;Alice∣Bob=employed,Tom=employed)=0.

These examples illustrate that CIR is an assessment of predictive necessity under specific observed conditions—it describes relationships in data, not absolute properties of variables or an underlying function.

### 3.2. Understanding Predictive Redundancy

We now consider a modified scenario. Suppose Bob and Alice both serve as accountants—they perform identical roles—and the company is operational as long as at least one is employed. From a management perspective, assuming at least one of them is working, they are interchangeable.

In this restricted domain, Bob and Alice are predictively redundant. Formally, it is as follows:

(15) I(Company;Bob)=I(Company;Alice)=I(Company;Bob,Alice)

This is a symmetric relationship: Bob is CIR given Alice’s presence, and Alice is CIR given Bob’s presence. This mutual conditional irrelevance is precisely what makes them predictively redundant—each can substitute for the other without information loss.

However, any assessment of PR depends on which cases are observed. In the scenario above, where at least one accountant is always employed, Bob and Alice are PR. But if management also observes cases where both are unemployed (and the company fails), the relationship changes. In that expanded dataset, neither Bob nor Alice is sufficient alone—both contribute to prediction, and PR no longer holds. Like CIR, PR is an assessment of relationships in observed data.

### 3.3. CIR and PR with Independent Inputs

The previous examples involved inputs that were statistically dependent within the observed contexts. Here, we demonstrate that CIR and PR can emerge even when inputs are pairwise independent. Consider the following dataset:

$$\begin{array}{cccc} X_{1} & X_{2} & X_{3} & Y\\ 0 & 0 & 0 & 0\\ 1 & 0 & 0 & 0\\ 0 & 1 & 0 & 0\\ 1 & 0 & 0 & 0\\ 0 & 1 & 0 & 0\\ 1 & 1 & 0 & 0\\ 0 & 0 & 1 & 0\\ 1 & 1 & 1 & 1 \end{array}$$

In this dataset, $X_{1}$, $X_{2}$, and $X_{3}$ are pairwise independent: $I\left(X_{1};X_{2}\right)=I\left(X_{2};X_{3}\right)=I\left(X_{1};X_{3}\right)=0$. Despite this independence, $X_{1}$ and $X_{2}$ exhibit both CIR and PR with respect to *Y* within the observed data.

This example demonstrates what the definitions stated: CIR and PR describe redundancy for prediction—whether inputs are interchangeable or conditionally unnecessary—not redundancy of informational content. Since $X_{1}$ and $X_{2}$ share no information with each other, any redundancy between them must be about their predictive roles, not about overlapping content.

### 3.4. Understanding Informational Redundancy

To understand what informational redundancy measures are, suppose there exist three books:

1. A collection of an author’s short stories;
2. A novel by the same author, which includes one of those stories as its opening chapter;
3. An anthology of stories by various authors in the same genre, which includes the same story.

The collection and the novel share content in the form of the identical chapter. This content is also shared with the anthology. This is a clear and intuitive notion of redundant information—if you have read the story in either the collection or the novel, you already know that content as it appears in the anthology. Critically, in this idealized case, the shared part is identifiable as a discrete object.

Now, imagine that the remaining pages in each work (outside the shared story) contain completely random text with no structure. In this case, there would be no correlation between the books aside from the chapter. The mutual information between any pair would equal exactly the entropy of the shared chapter, and we could precisely identify and quantify the common information. This is precisely the scenario that classical common-information measures like Gács–Körner handle well: deterministic shared structure that can be cleanly extracted and represented.

However, in reality, correlations extend beyond the obviously shared chapter. The collection and the novel are by the same author, so they exhibit statistical regularities—characteristic word choices, sentence structures, thematic patterns—that create additional mutual information. Similarly, since the anthology contains stories of the same genre, it too exhibits statistical correlations with the other works beyond the explicit chapter overlap.

This presents a fundamental challenge. The shared chapter represents identifiable common content that can, in principle, be extracted and represented as a classical object. The statistical correlations, however, are real—they contribute to I(Collection,Novel)—but cannot be cleanly separated and represented as a distinct random variable. They are diffuse, distributed across the structure of the texts.

Both forms of shared content contribute to informational redundancy. The idealized framework introduced earlier addresses this by defining common information not through classical extraction, but through information-theoretic constraints. The common-information object C(Collection,Novel) is defined to contain all the information shared between them—both the explicit chapter and the distributed statistical regularities—even though no classical random variable can represent this completely.

The idealized common-information framework objectifies this shared content as C(Collection,Novel)—an object that contains both the explicit chapter and the more tenuous correlations that exist only as distributed statistical patterns. To determine informational redundancy, we must identify which portion of this shared content is relevant to the Anthology. This is accomplished by filtering the common information through its relationship with the target: C(C(Collection,Novel),Anthology). This captures only the shared content between Collection and Novel that also appears in Anthology—in this case, primarily the chapter they all contain.

More generally, in information decomposition, IR measures how much of I(X;Y) arises from such shared content among inputs. When inputs share both obvious structure and subtle statistical patterns, IR captures the total contribution of that overlap to predicting the output.

## 4. Discussion

The examples presented above illustrate a fundamental distinction between operational and informational notions of redundancy. CIR and PR describe predictive relationships in observed data—whether inputs are conditionally necessary or interchangeable for prediction. As demonstrated, both can emerge even when inputs share no information with each other. IR, by contrast, measures shared informational content itself—content that multiple inputs hold in common and that contributes to the output.

This reveals an apparent unavoidable constraint: only IR is grounded in mutual information between inputs, and therefore of the forms of redundancy we have discussed, only IR is suitable for inclusion as a term in decompositions of I(X;Y).

### 4.1. What Qualifies as a Mutual Information Decomposition Term?

A decomposition of mutual information must contend with what is being decomposed: the correspondence between input states and output states. Mutual information I(X;Y) quantifies this correspondence—how the state of *X* constrains the state of *Y*, and vice versa. Any term in a decomposition of I(X;Y) must, therefore, be grounded in this state correspondence structure.

For a redundancy term specifically, this imposes a requirement. Consider the book example: the collection and novel contain informational redundancy because the same chapter—the same content—appears in both. This overlap in content is what allows for redundancy with respect to the Anthology. I(Collection;Novel)>0 quantifies the existence of shared structure between Collection and Novel, a requirement if we are to say that they participate in redundancy in a meaningful informational sense.

Now, consider what happens when $I(X_{i};X_{j})=0$. This means the states of $X_{i}$ tells you nothing about the states of $X_{j}$—they vary independently. In terms of content, whatever information $X_{i}$ carries is entirely distinct from whatever information $X_{j}$ carries. They are like two books with no shared chapters, no common passages, no overlapping text.

Redundant information requires the same content to appear in multiple places. When $I(X_{i};X_{j})=0$, there is no “same content” between $X_{i}$ and $X_{j}$. Each may inform *Y*, but they must be doing so through entirely different content. Operational redundancy need not involve redundant information.

The confusion arises because $X_{i}$ and $X_{j}$ might both predict *Y* equally well (PR) or might each become unnecessary when the other is known (CIR). These do identify a relationship that is in fact redundancy, just not of an informational type.

### 4.2. Why Confusion About “What Redundancy Is” Persists

The persistent difficulty in defining redundancy stems from a fundamental ambiguity in what “redundancy” means. In ordinary usage, redundancy refers to multiple distinct phenomena, for example:

- Backup redundancy: Having multiple systems that can substitute for each other (PR);
- Unnecessary redundancy: Having components that become superfluous under certain conditions (CIR);
- Duplication redundancy: Having the same content appear in multiple places (IR).

All three intuitions are valid. All three describe real structures in systems. The problem arises when we attempt to formalize “redundancy” as a single term in an information decomposition without first distinguishing which notion we mean.

CIR and PR capture important intuitions about redundancy—intuitions that arise naturally when examining how systems behave, how predictions are made, and how components interact. It is entirely reasonable that researchers have sought to incorporate these intuitions into information-theoretic frameworks. The difficulty is that these operational properties do not correspond to components of I(X;Y) in the way that informational redundancy must.

This explains why multiple incompatible redundancy measures have emerged, each satisfying different axioms and producing different results. The measures are not simply competing approximations of the same underlying quantity. They are formalizing different concepts that have been conflated under a single term. No single measure can satisfy all these intuitions simultaneously because the intuitions themselves are about different things.

## 5. Engagement with Existing PID Measures

Having formalized the distinction between operational and informational redundancy, we now examine how existing PID measures relate to this framework. Our analysis reveals that the majority of PID redundancy proposals measure forms of operational redundancy—they quantify predictive properties such as sufficiency, substitutability, or necessity, rather than shared informational content among inputs. In only one measure we examine—the source redundancy term of Pica et al.—is informational redundancy explicitly addressed.

Importantly, while we can categorize measures as operational or informational, we cannot precisely map most operational measures to the specific examples (CIR or PR) we formalized in Section 2. The operational class is broad, encompassing many possible formalizations of predictive relationships. CIR and PR are illustrative representatives, not exhaustive. Each PID measure operationalizes its own criterion for what constitutes operational redundancy—whether through specific information, minimum mutual information, conditioning strategies, or distributional constraints. What these measures share is that they can detect predictive relationships that do not require mutual information between inputs, and therefore measure operational properties rather than content overlap.

Below, we characterize several widely used measures to demonstrate how the conceptual ambiguity identified in Section 2 manifests across the literature.

### 5.1. $I_{min}$

**Definition** **1.**
(16) $$I_{\min}\left(X_{1},X_{2};Y\right)=\sum_{y\in Y}P_{Y}\left(y\right)\min_{i}I_{\mathrm{spec}}\left(X_{i};y\right),$$

*where $I_{\mathrm{spec}}\left(X_{i};y\right)$ is the specific information provided by $X_{i}$ about the output state y [4].*

Interpretation: $I_{\min}$ defines redundancy by comparing how informative each source is about individual output states. For each *y*, it selects the source that provides the least specific information about that outcome, and then averages this quantity over *y*. Redundancy is thus identified with the amount of predictive information that is guaranteed to be available regardless of which source is consulted.

Because this construction evaluates sources independently for each output state and does not depend on shared structure between the inputs, $I_{\min}$ can be positive even when $I(X_{1};X_{2})=0$. From the perspective adopted here, $I_{\min}$ therefore represents operational redundancy based on source substitutability, rather than redundancy grounded in shared informational content, which has been noted in prior literature [6,16].

### 5.2. $I_{MMI}$ (Minimum Mutual Information)

**Definition** **2.** *The minimum mutual information (MMI) redundancy defines redundancy as follows:*

(17) $$I_{\mathrm{MMI}}\left(X_{1},X_{2};Y\right)=\min\left\{I\left(X_{1};Y\right),\;I\left(X_{2};Y\right)\right\}.$$

*Redundancy is identified with the smallest amount of information that any single source provides about the target [17].*

Interpretation: This construction defines redundancy as a guaranteed level of predictability: the amount of information about *Y* that is available regardless of which source is observed. The measure does not depend on correlations or shared structure between the inputs, but only on their individual predictive relationships with the target.

From the perspective adopted here, $I_{\mathrm{MMI}}$ therefore represents operational redundancy based on worst-case source substitution. It quantifies overlap in predictive capability rather than redundancy grounded in shared informational content among the inputs.

### 5.3. $I_{sx}$

**Definition** **3.** *$I_{sx}$ defines redundancy by conditioning on the event that at least one source realizes a designated state. For two binary sources, redundancy is identified with the information that the event $\left(X_{1}=1\right)\vee \left(X_{2}=1\right)$ provides about the target Y [3].*

This construction defines redundancy relative to a task-specific conditioning on valid cases, corresponding to predictive sufficiency under the assumption that any one of the sources is sufficient. From the perspective adopted here, $I_{sx}$ therefore represents operational redundancy, rather than redundancy grounded in shared informational content among the inputs.

### 5.4. $I_{BROJA}$

**Definition** **4.** *Unique information is defined as follows:*

(18) $$UI\left(X_{1}\backslash X_{2}\right)=\min_{Q\in \Delta }I_{Q}\left(X_{1};Y|X_{2}\right),$$

*where Delta is the set of joint distributions on $\left(X_{1},X_{2},Y\right)$ that preserve the marginal distributions $P(X_{1},Y)$ and $P(X_{2},Y)$. Redundancy is then defined implicitly as $I\left(X_{1};Y\right)-UI\left(X_{1}\backslash X_{2}\right)$ [8].*

Interpretation: This construction defines redundancy relative to a constrained optimization over alternative joint couplings of the sources. By fixing the source–target marginals and minimizing conditional information, $I_{\mathrm{BROJA}}$ identifies the portion of information that cannot be uniquely attributed to one source once the other is taken into account, under the least favorable coupling consistent with the observed marginals.

Redundancy is thus characterized in terms of predictive non-uniqueness under marginal constraints, rather than in terms of shared informational content intrinsic to the observed joint distribution. From the perspective adopted here, $I_{\mathrm{BROJA}}$ therefore represents operational redundancy based on counterfactual source substitutability, rather than redundancy grounded in shared informational content among the inputs.

### 5.5. Source vs. Non-Source Redundancy

**Definition** **5.** *Pica et al. distinguish between source redundancy (SR), which is bounded by $I(X_{1};X_{2})$ and attributed to correlations between inputs, and non-source redundancy (NSR), defined as the remainder of the total redundancy assigned by a chosen PID measure and attributed to the mechanism [18].*

Interpretation: This distinction explicitly separates redundancy arising from shared structure in the inputs from redundancy arising through the input–output mapping. In particular, depending on the chosen PID redundancy measure, NSR can be nonzero even when $I(X_{1};X_{2})=0$, as in the case of an AND gate with independent inputs, where different input states independently predict the same output.

From the perspective adopted here, Pica et al.’s source redundancy corresponds to informational redundancy insofar as both concern shared content between inputs. However, Pica et al.’s SR is constructed operationally by comparing redundancy atoms across different PID lattices (with different target assignments), rather than by extracting common information. While SR is bounded by $I(X_{1};X_{2})$ and increases when source correlations contribute to redundancy, it does not isolate a common-information object as defined in Section 2.2.2. Instead, SR quantifies the portion of operational redundancy that could be attributed to source correlations. Non-source redundancy (NSR), by contrast, captures predictive overlap that occurs independently of source correlations—a purely operational phenomenon. Thus, while the SR/NSR split acknowledges the role of source correlations, it remains within the operational paradigm of PID and does not provide a true measure of shared informational content.

### 5.6. Local PID Measures

**Definition** **6.** *Local PID measures define redundancy at the level of individual realizations by decomposing pointwise mutual information i(x;y) into local redundant, unique, and synergistic contributions. These quantities characterize how particular observations of the inputs contribute information about a specific outcome of the target [6].*

Interpretation: Local information-theoretic analysis shifts the object of study from the ensemble distribution to distributions conditioned on particular realizations. Conditioning on an event (x,y) induces a new informational structure, with its own dependencies and overlaps, which local PID measures then decompose. In this sense, local measures do not identify the location of pre-existing shared informational content, but rather characterize the informational structure of the conditioned world associated with a specific observation.

From the perspective adopted here, local PID therefore captures event-level predictive overlap: cases in which different inputs independently or jointly support the same prediction for a given outcome. While this provides valuable insight into the informational role of individual realizations, it does not define informational redundancy as shared content among inputs at the ensemble level. When local redundancy measures are averaged over the joint distribution to obtain ensemble quantities, the resulting measures correspond to operational redundancy rather than redundancy grounded in shared informational content.

### 5.7. Information-Geometric Redundancy

**Definition** **7.** *Harder et al. define redundancy using tools from information geometry. Redundancy is identified by comparing the conditional distributions $P(Y|X_{1})$ and $P(Y|X_{2})$, and measuring the extent to which these conditionals are equivalent under projection onto a common information-geometric manifold. Intuitively, redundancy is associated with overlap in how different sources constrain beliefs about the target [19].*

Interpretation: This construction characterizes redundancy in terms of equivalence classes of predictive distributions: two sources are redundant to the extent that they induce the same posterior beliefs about *Y*. The measure does not depend on shared structure or correlation between the inputs themselves, but on similarity in their predictive effects on the target.

From the perspective adopted here, the information-geometric approach therefore represents operational redundancy. It quantifies when different sources lead to the same predictions about the target, regardless of whether those predictions arise from shared informational content or from independent mechanisms. The use of geometric projection provides a principled way to identify predictive equivalence, but the resulting redundancy reflects overlap in predictive implications rather than shared information among the inputs.

### 5.8. Summary

The variety of redundancy measures proposed within the PID framework reflects a fundamental ambiguity about what redundancy is intended to capture. Existing measures operationalize different criteria for when sources should be regarded as providing the same contribution to a target, leading to formally incompatible notions of redundancy.

All the PID redundancy measures discussed here instantiate forms of operational redundancy (with the partial exception of source redundancy). These quantities characterize overlap in the predictive or inferential role of different sources—such as substitutability, guaranteed predictability, or equivalence of induced posteriors. Such quantities are operationally meaningful and widely useful, and their prevalence in applications is well motivated; however, the information they specify need not respect decompositional boundaries and can span multiple terms in a Shannon-based decomposition.

Informational redundancy, by contrast, concerns shared informational content and requires a criterion for when multiple sources provide the same information about a target. This notion is distinct in kind from operational redundancy, clarifying why existing PID measures disagree and why no single redundancy measure can satisfy all intuitions simultaneously.

## 6. Conclusions

This paper has argued that the long-standing difficulty in defining redundancy is primarily conceptual rather than technical. In particular, the term “redundancy” has been used to refer to two fundamentally different classes of phenomena: one relating to predictive capacity and the other to shared informational content. Much of the disagreement in the literature arises from conflating these classes under a single label.

To clarify this conceptual space, we formalized the two classes of redundancy using examples. For operational redundancy, we described conditional irrelevance (CIR) and predictive redundancy (PR), which each capture different aspects of predictive capacity—whether an input is necessary under conditioning, and whether inputs are interchangeable for prediction, respectively. The other form, informational redundancy (IR), by contrast, concerns shared informational content among inputs that contributes to the output. These classes are not alternative measures of a single quantity, but formalizations of distinct questions about necessity, sufficiency, and content overlap.

The definitions of redundancy presented here are not meant to be exhaustive. We selected these particular concepts because they capture the most common sources of confusion in the literature and in colloquial usage, but the space of relationships that might reasonably be called “redundancy” is richer than any simple taxonomy. Other definitions can coexist alongside ours (for example, predictive equivalence: where inputs contribute equally to prediction), and different framings might carve the conceptual space differently. What matters is not the particular classification scheme, but the recognition that redundancy refers to two fundamentally distinct classes of phenomena. The relationships these concepts formalize are real; our labels and groupings are tools for navigating that complexity.

These distinctions between CIR and PR on the one hand and IR on the other have logical consequences for information decomposition. A decomposition of mutual information I(X;Y) must partition that mutual information into non-overlapping components measured in bits. For a redundancy term to represent a component of such a decomposition, it must account for some portion of that mutual information. IR satisfies this requirement because it depends on shared structure between inputs: without mutual information between $X_{i}$ and $X_{j}$, there is no shared informational content that could contribute to I(X;Y). CIR and PR do not satisfy this requirement—both can exist when $I(X_{i};X_{j})=0$. When inputs are independent, the total information I(X;Y) must decompose into only unique and synergistic components, yet CIR and PR remain definable and detectable.

This demonstrates that CIR and PR are orthogonal to information-theoretic decomposition. They describe operational relationships between inputs and outputs that exist independently of whether the underlying information is unique, synergistic, or redundant. While CIR and PR may represent proper forms of redundancy—inputs appearing interchangeable or conditionally unnecessary—this is not the sort of redundancy that can participate in a decomposition. What they quantify is not contingent on shared content. As such, their detection is not a reliable indicator of redundant information in a decomposition, as they are not measures of informational content.

Functional Information Decomposition (FID) [20], introduced in related work, provides an alternative approach that generates information decompositions without needing to resolve common information or introduce redundancy as a decomposition component. FID operates on complete functional specifications in which inputs vary independently over their full Cartesian product. Under these conditions, $I(X_{i};X_{j})=0$ by construction, making informational redundancy impossible—IR equals zero by definition. Under FID, decomposition includes only independent and synergistic components. When observations are partial, FID samples the space of possible completions, generating distributions over decompositions that explicitly represent what the data determine and what remains uncertain. This approach inverts the common information problem: rather than seeking an idealized object that captures all shared content, FID enumerates concrete completions that respect observational constraints.

Alternatively, frameworks may choose to treat CIR and PR as complementary analyses that characterize system behavior without claiming to partition I(X;Y). Both approaches—decomposition that excludes CIR/PR, and operational analysis that uses them—are valid. The confusion has arisen from conflating them.

The path forward requires being precise about what question is being asked. If the question is “how do inputs share content that informs the output?”, the answer is IR. If the question is “when are inputs interchangeable for prediction?”, the answer is PR. If the question is “when do inputs become unnecessary under conditioning?”, the answer is CIR. And there are other questions along these lines that one could ask. These are different questions with different answers, and attempting to force them into a single “redundancy” measure has obscured rather than clarified the structure of multivariate information.

## Data Availability

No new data were created or analyzed in this study.

## Abbreviations

The following abbreviations are used in this manuscript:

CIR	Conditional Irrelevance
CI	Conditional Independence
PR	Predictive Redundancy
IR	Informational Redundancy
PID	Partial Information Decomposition
FID	Functional Information Decomposition

## References

[1] McGill W.J. (1954). Multivariate information transmission. Psychometrika.

[2] Watanabe S. (1960). Information theoretical analysis of multivariate correlation. IBM J. Res. Dev..

[3] Makkeh A., Gutknecht A.J., Wibral M. (2021). Introducing a differentiable measure of pointwise shared information. Phys. Rev. E.

[4] Williams P.L., Beer R.D. (2010). Nonnegative decomposition of multivariate information. arXiv.

[5] Griffith V., Koch C., Prokopenko M. (2014). Quantifying synergistic mutual information. Guided Self-Organization: Inception.

[6] Finn C., Lizier J.T. (2018). Pointwise partial information decomposition using the specificity and ambiguity lattices. Entropy.

[7] Kolchinsky A. (2024). Partial Information Decomposition: Redundancy as Information Bottleneck. Entropy.

[8] Bertschinger N., Rauh J., Olbrich E., Jost J., Ay N. (2014). Quantifying unique information. Entropy.

[9] Griffith V., Chong E.K.P., James R.G., Ellison C.J., Crutchfield J.P. (2014). Intersection information based on common randomness. Entropy.

[10] Rosas F.E., Mediano P.A.M., Gastpar M., Jensen H.J. (2019). Quantifying high-order interdependencies via multivariate extensions of the mutual information. Phys. Rev. E.

[11] Bohm C., Kirkpatrick D., Cao V., Adami C. (2022). Information Fragmentation, Encryption and Information Flow in Complex Biological Networks. Entropy.

[12] Gács P., Körner J. (1973). Common information is much less than mutual information. Probl. Control Inf. Theory.

[13] Wyner A.D. (1975). The common information of two dependent random variables. IEEE Trans. Inf. Theory.

[14] Tishby N., Pereira F.C., Bialek W. (2000). The information bottleneck method. Proceedings of the 37th Annual Allerton Conference on Communication, Control, and Computing.

[15] Rota G.-C. (1964). On the foundations of combinatorial theory I: Theory of Möbius functions. Z. Wahrscheinlichkeitstheorie Verwandte Geb..

[16] Kay J.W., Schulz J.M., Phillips W.A. (2022). A Comparison of Partial Information Decompositions Using Data from Real and Simulated Layer 5b Pyramidal Cells. Entropy.

[17] Kay J.W., Ince R.A.A. (2018). Exact Partial Information Decompositions for Gaussian Systems Based on Dependency Constraints. Entropy.

[18] Pica G., Piasini E., Chicharro D., Panzeri S. (2017). Invariant components of synergy, redundancy, and unique information among three variables. Entropy.

[19] Harder M., Salge C., Polani D. (2013). A bivariate measure of redundant information. Phys. Rev. E.

[20] Bohm C., Ragusa V.R., Hintze A., Adami C. (2025). Functional Information Decomposition: A First-Principles Approach to Analyzing Functional Relationships. arXiv.

